# The impact of pipeline changes and temperature increase in a hospital historically colonised with *Legionella*

**DOI:** 10.1038/s41598-021-81625-6

**Published:** 2021-01-21

**Authors:** S. Quero, N. Párraga-Niño, M. Garcia-Núñez, M. L. Pedro-Botet, L. Gavaldà, L. Mateu, M. Sabrià, J. M. Mòdol

**Affiliations:** 1 Infectious Diseases Unit, Fundació Institut d’Investigació Germans Trias I Pujol, Carretera de Can Ruti, Camí de les Escoles s/n, 08916 Badalona, Barcelona Spain; 2 CIBER de Enfermedades Respiratorias, CIBERES, Av. de Monforte de Lemos, 3-5, 28029 Madrid, Spain; 3 Institut d’Investigació i Innovació Parc Taulí, I3PT, Parc Taulí, 1, 08208 Sabadell, Barcelona Spain; 4 Hospital Universitari Germans Trias i Pujol, Carretera de Canyet, s/n, 08916 Badalona, Barcelona Spain; 5 Hospital Universitari de Bellvitge-IDIBELL, Feixa Llarga, s/n., 08907 L’Hospitalet de Llobregat, Barcelona Spain

**Keywords:** Water microbiology, Policy and public health in microbiology

## Abstract

Healthcare-related Legionnaires’ disease has a devastating impact on high risk patients, with a case fatality rate of 30–50%. *Legionella* prevention and control in hospitals is therefore crucial. To control *Legionella* water colonisation in a hospital setting we evaluated the effect of pipeline improvements and temperature increase, analysing 237 samples over a 2-year period (first year: 129, second year: 108). In the first year, 25.58% of samples were positive for *Legionella* and 16.67% for amoeba. Assessing the distance of the points analysed from the hot water tank, the most distal points presented higher proportion of *Legionella* colonisation and lower temperatures (nearest points: 6.4% colonised, and temperature 61.4 °C; most distal points: 50% and temperature 59.1 °C). After the first year, the hot water system was repaired and the temperature stabilised. This led to a dramatic reduction in *Legionella* colonisation, which was negative in all the samples analysed; however, amoeba colonisation remained stable. This study shows the importance of keeping the temperature stable throughout the circuit, at around 60 °C. Special attention should be paid to the most distal points of the circuit; a fall in temperature at these weak points would favour the colonisation and spread of *Legionella*, because amoeba (the main *Legionella* reservoir) are not affected by temperature.

## Introduction

*Legionella* spp. is the causative agent of Legionnaires’ Disease (LD)^1–4^. Specifically, *Legionella pneumophila* is associated with the majority of LD cases^1–5^. LD is an atypical pneumonia caused by the inhalation of aerosols containing *Legionella*. Hospital-acquired LD has been reported to be responsible for up to 14% of cases of health-care associated pneumonia^6,7^. In 2015 the European Legionnaires’ Disease Surveillance Network (ELDSNet) reported that 8% of LD cases were related to healthcare facilities^8^. The fatality rate of LD is about 10%, but this figure rises to 30–50% in hospital-acquired LD; therefore, the prevention and control of *Legionella* in hospitals is essential^9^.

The main reservoirs of *Legionella* are water-related natural habitats, where it is known to form biofilms in surface interphases or to survive within amoeba. *Legionella* can colonise man-made water distribution systems, where it multiplies; indeed, these sources have been associated with LD cases and outbreaks^10–14^. In hospitals, cold and hot water distribution systems are the main sources of infection^9,15,16^. The factors that affect *Legionella* colonisation and growth are the temperature and physicochemical parameters of the water, stagnation, the material used in the pipelines, and the pipelines’ age^9,17–19^. The ability to survive and grow in biofilm and the presence of parasitising amoebas are other important factors in *Legionella* colonisation.

Prevention and control of *Legionella* colonisation in health-care units is important because many hospitalised patients have a high risk of *Legionella* infection. The most effective methods for *Legionella* disinfection in water distribution systems are chlorination, heating, and copper-silver ionisation^3,20^. According to the World Health Organisation (WHO) guidelines and Spanish (RD865/2003) and Catalan (DOGC352/2004) legislation, hot water systems should be maintained at 50 °C in the most distal points and at 60 °C in hot water tanks^15,21,22^. Furthermore, the Catalan regulations stipulate that complementary measures such as superheating or hyperchlorination flushing should be performed if periodical *Legionella* control in the water distribution system identifies more than 30% of points as positive.

The objective of this study was to evaluate the effectiveness of hot water system replacement and temperature increase to prevent *Legionella* and amoeba colonisation.

## Results

### Environmental surveillance

Of the 237 samples collected, 33 were positive for *Legionella* (13.92%) and 29 out of 180 samples analysed were positive for amoeba (16.11%). *L. pneumophila* sg 2–15 was recovered in all but four positive samples. Two samples were colonised by *L. anisa*, one by *L. pneumophila* sg 1 and one by a mix of *L. pneumophila* sg 1 and *Legionella* no-*pneumophila*.

### Results according to intervention period—first year (observational period)

From 129 samples tested for *Legionella* presence during the first year, 33 were positive (25.58%), while 18 out of 108 samples in the amoeba analysis were positive (16.67%). The mean temperature was 61.4 °C (95CI% 60.19–61.28). Sample temperature ranged from 40.7 to 65.5 °C, indicating a high variability in the hot water system.

#### Temperature level and colonisation

In order to assess the importance of temperature and the presence of *Legionella* and amoeba, the samples of the first year were classified according to a 60 °C cut-off. Thirty-seven out of 129 samples were below 60 °C (28.68%) and 31 were positive for *Legionella* (54.05%); in contrast, only 13 samples out of 92 (14.13%) above 60 °C were positive for *Legionella* (p < 0.0001). Amoeba colonisation was not affected by the sample temperature (19.35% of samples < 60 °C and 15.58% of samples > 60 °C were colonised by amoeba). There was no correlation between amoeba and *Legionella* colonisation.

#### Influence of the distance from the hot water tank and temperature variation

**Figure 1:**
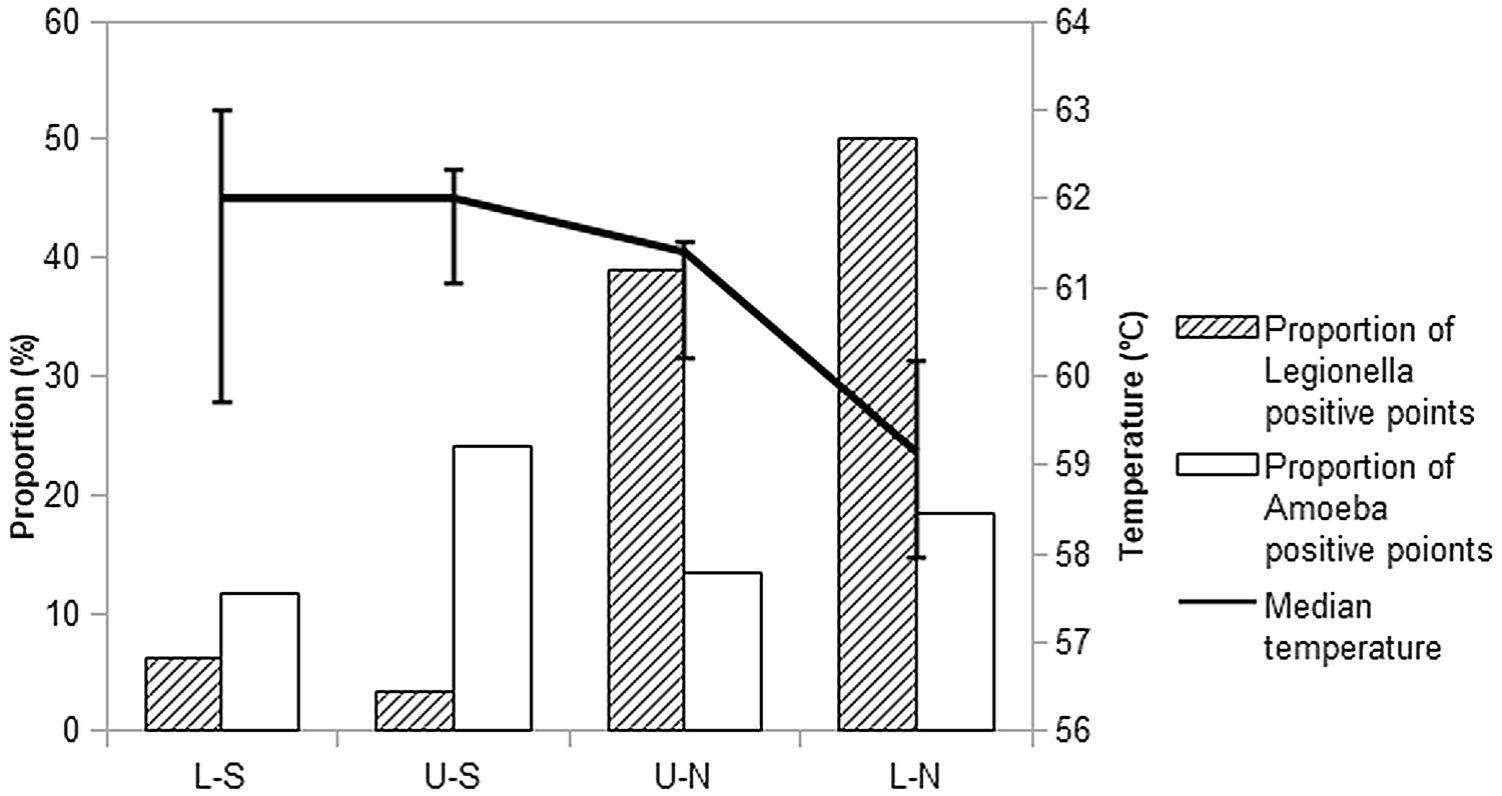
Variations of temperature, Legionella and amoeba colonisation during the observational period (first year) according to the distance from the hot water tank, as defined by the four areas: Lower-South (L-S), Upper-South (U-S), Upper-North (U-N) and Lower-North (L-N).

Comparison of the four areas during the first year showed that the further from the hot water tank, the lower the temperature. The mean temperature was 62 °C (95%CI 59.7–63) in the Lower-South area and 59.15 °C (95%CI 57.97–60.16) (p < 0.0001) in the Lower-North area (Fig. 1). The water temperature fell in the two areas facing north (South: 62.73 °C (95%CI 61.9–63.5), North: 58.52 °C (95%CI 58.3–58.8); p < 0.0001). The increase in points colonised by *Legionella* was associated with the distance from the hot water tank as a result of the fall in temperature (south: 4.92% *Legionella-*positive points, north: 44.12% *Legionella-*positive points; p < 0.0001). Amoeba colonisation remained stable throughout the building, and was not affected by the fall in temperature.

### Results according to intervention period—second year

**Figure 2:**
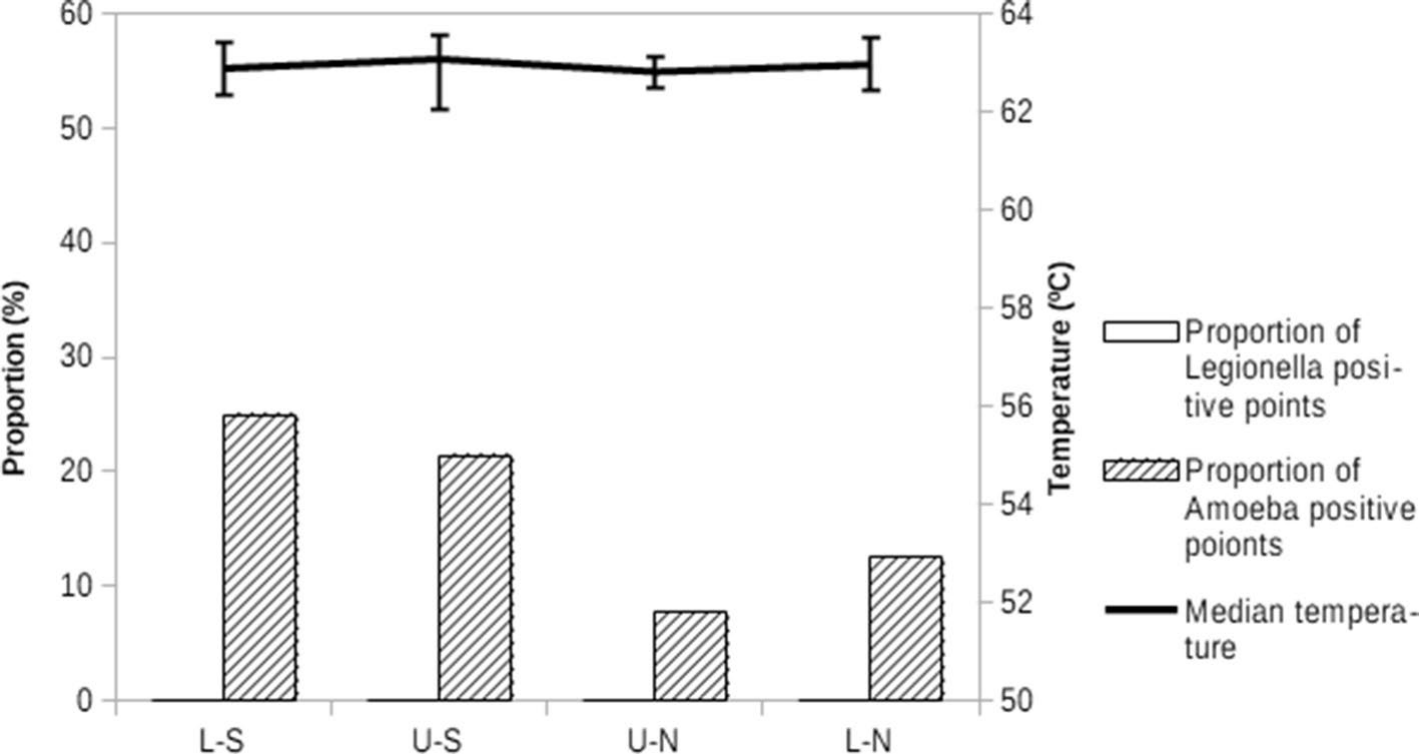
Variations of temperature, Legionella and amoeba colonisation after the implementation of the new measures (temperature increase and change of pipeline material, second year) according to the distance from the hot water tank, defined by the four areas: Lower-South (L-S), Upper-South (U-S), Upper-North (U-N) and Lower-North (L-N).

After the technical changes, the mean temperature was 62.95 °C (95%CI 62.7–63.15, range: 59.8 °C to 65.9 °C). Only one sample was below 60 °C. Furthermore, the temperature was kept stable in the four areas of the building (Fig. 2). None of the samples were positive for *Legionella*, although 15.28% (11/72) were positive for amoeba; there were no differences between the points nearest and furthest away from the hot water tank (Fig. 2).

### Clinical surveillance

During the 2-year study period, no cases of hospital-acquired LD were diagnosed.

## Discussion

Large buildings with hot water tank, like hospitals, health-care units and hotels are susceptible to colonisation by *Legionella.* In these buildings the temperature must be kept high in order to prevent *Legionella* colonisation and cases of LD. The WHO guidelines and Spanish and Catalan legislation recommend maintaining temperatures of at least 50 °C in circulating hot water and above 60 °C in the hot water tank; however, the WHO fact sheet states that at 50 °C *Legionella* is able to persist and colonise the pipeline system^23^.

The hospital where this study was performed has a past history of *Legionella* colonisation. Several disinfection measures were applied, but none were able to completely eradicate the bacterium^24,25^. The intervention described here to eliminate *Legionella* colonisation showed the importance of the pipeline material and of maintaining the water at a temperature of 60 °C in the distal points. The points below 60 °C were the ones that presented the highest rates of colonisation.

Before the replacement, the water temperature in the pipeline in the area nearest the hot water tank was around 60 °C but the temperature was lower at points further away: most of the samples below 60 °C were from the Lower-North area, the one furthest away from the tank. The larger a suboptimal pipeline system is, the greater the heat loss. Although the temperature of circulating water started at 60 °C, this level was not maintained across the entire pipeline network, and at the points with lower temperatures *Legionella* was able to colonise the site and persist inside the circuit.

In the second year, increasing the temperature and changing the pipeline system (i.e., replacing galvanised iron with PVC) achieved a stable temperature throughout the building, with all but one sample remaining above 60 °C. This measure was able to eradicate the *Legionella* colonisation in the building; however, since amoeba are still present in the pipeline and we did not evaluate the VBNC *Legionella*, the risk of pipeline *Legionella* re-colonisation remains if the temperature falls once more.

Corrective measures and thermal regimes have been implemented in large buildings to eradicate *Legionella* from water systems. Gavaldà et al*.* concluded that a combination of corrective measures and raising the temperature at the end points to 55 °C might reduce the presence of *Legionella*^26^. However, after implementing the same measures, Bédard et al*.* achieved a reduction of *Legionella* colonisation in only one of the two systems analysed^27^.

Maintaining the temperature of the circulating water above 60 °C entails a high energy cost. An alternative measure for preventing LD cases in hospital is the installation of point-of-use filters in the faucets. This measure is currently in use in high risk patient areas, but it is expensive and effective only at the installed points; what is more, its effects are only transitory. The installation of point-of-use filters in areas with high risk patients entails a cost of nearly 30 000€/year (for 100 points), whereas raising the temperature in the return pipe to the hot water tank from 50 to 55 °C would suppose an increase of around 32 000€/year. So, the cost is similar, but raising the temperature eradicates *Legionella* throughout the building—not just at the most susceptible points.

One way to reduce the cost of keeping the circulating water above 60 °C would be to install local heaters in critical areas. This would mean that the temperature in the tank would not have to be 60 °C in order to keep the circuit at the level required; it would only be necessary to increase the temperature at the “weak” points. Local heaters would drastically bring down the energy costs of heating the tank to high temperatures, and would also avoid overheating of the pipeline, minimise pipeline damage, and reduce the risk of scalding patients.

Local heaters are ideal for buildings with large pipelines and with numerous distal points, such as the one analysed here (with 13 floors and 28 rooms on each floor). These local heaters would also be a good solution for old buildings which use hot water tanks and recirculating water where *Legionella* can persist in the pipeline, and where colonisation has not been prevented by the technical changes carried out. As the WHO suggested, future buildings should be built using a pipeline material that inhibits biofilm formation, and should implement disinfection measures at the beginning of water circulation to prevent *Legionella* colonisation^28^. Furthermore, temperature control studies should be conducted to identify critical areas where *Legionella* can grow, and local heaters should be installed to avoid its proliferation.

In the study by Lasheras et al*.*, another factor shown to correlate with *Legionella* colonisation was water hardness. Those authors concluded that softer water was better in this situation^29^. As our hospital’s water is semi-hard^30^, it would be advisable to install a water softener to improve water quality.

Though *Legionella* was undetectable in the system after the pipeline improvement, amoebas were not affected by this change. Lasheras et al*.* found a correlation between the presence of amoebas and a larger number of *Legionella*-positive points, since amoebas are *Legionella* reservoirs^29^. Although we did not detect *Legionella* in the second year, we cannot be entirely sure that it was not present in the pipeline; after environmental stresses, *Legionella* can change its metabolic state to viable but non-culturable (VBNC) and settle in biofilms or parasite amoebas. In any of these states, *Legionella* is more difficult to detect. In the VBNC state *Legionella* has low metabolic activity and does not grow in culture media, but in spite of this low growth rate the bacteria retain the features of viable cells such as their integrity and virulence^31,32^.

The impossibility of growing bacteria in VBNC in culture media is a limitation both of our study and of the current regulations regarding environmental *Legionella* control. The absence of culturable *Legionella* in water distribution systems does not guarantee water healthiness/safety because VBNC *Legionella* maintains its capacity to infect and to cause hospital-acquired LD in the most vulnerable/susceptible patients. In this situation there is a clear need to change the current regulations, and to add the detection of VBNC to the culture plate method currently in use. VBNC can be identified by the detection of specific genetic material with PCR using reagents such as propidium monoazide (PMA) or ethidium monoazide bromide (EMA), by antibody detection using flow cytometry protocols, or by reactivation of *Legionella* cells by adding activating agents or infecting amoeba cells^32–34^. The use of these methods in environmental *Legionella* control would help to broaden our understanding of *Legionella* colonisation in water distribution systems. They would also provide information on the power of the disinfectants used in the facility, and on their modes of action.

## Conclusions

In this study we evaluated the colonisation of *Legionella* and amoeba in a hospital water distribution system, before and after the implementation of two technical changes: the replacement of the pipeline material in circuit 2, and the increase in the temperature of the hot water tank. A temperature of 55 °C was achieved at the end of the circuit, just before the water returns to the tank. Moreover, the water temperature in the four areas of the hospital was stable. After the changes, a decrease in *Legionella* colonisation was observed, while amoeba colonisation remained unaltered. Although the temperature increase is expensive, it improves *Legionella* control and reduces the likelihood of hospital-acquired LD. Local heaters and water softeners would be a good alternative for reducing the cost of maintaining the temperature required throughout the entire pipeline. These measures would also avoid the use of disposable filters in high-risk-patient areas, reducing both waste and the time needed for their replacement every month. Although *Legionella* was undetectable after an increase in temperature, efforts are now needed to reduce amoeba colonisation so as to avoid *Legionella* re-infection. The presence of *Legionella* in the VBNC state should also be evaluated before water can be considered *Legionella*-free.

## Material and methods

### Setting

**Figure 3:**
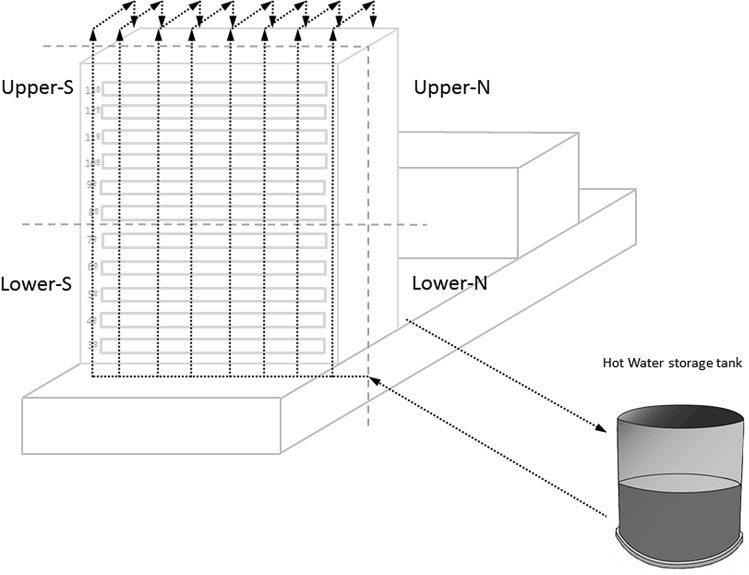
Diagram of hot water circuit 2. The arrows represent the direction of the water. The building is divided into four areas: Lower-S (Lower-South), Upper-S (Upper-South), Upper-N (Upper-North) and Lower-N (Lower-North).

The building analysed is a university hospital with 600 beds. This hospital includes renal and bone marrow transplantation programs and houses oncological and haematological wards. The hot water system has two independent water circuits which are fed by a single hot water tank. Circuit 1 supplies the three lowest floors (basement and first and second floors). Circuit 2 supplies floors 3 to 13, which house hospitalisation wards. The water from the hot water tank is distributed to circuit 2 as displayed in Fig. 3. The water from the returning pipes is re-heated and stored in the hot water storage tank, and then redistributed around the building according to requirements. I this study we evaluated the colonisation of circuit 2.

### Legionella control program

The disinfection methods used in the hot water system are copper-silver ionisation and heat-and-flush three times yearly. During the study period the levels of copper and silver ions were maintained at 0.2–0.4 ppm and 0.02–0.045 ppm respectively.

For sample analysis, the building was divided into four different areas according to the distance from the hot water tank. The nearest area to the tank was Lower-South (rooms facing south from floors 3 to 8), followed by Upper-South (rooms facing south from floors 9 to 13) and Upper-North (floors facing north from floors 9 to 13). Lower-North (rooms facing north from floors 3 to 8) was the area furthest from the hot water tank (Fig. 3).

### Study design

In order to quantify the effectiveness of the modifications, a before-and-after-changes study was designed. The first year was an observational period prior to the implementation of new measures to eradicate *Legionella* colonisation. After the observational period, two technical changes were carried out in the pipeline system of circuit 2 to improve *Legionella* control: the circuit’s galvanised iron pipes were replaced by PVC pipes, and the temperature in the hot water tank was raised in order to achieve a temperature of 55 °C in the return pipe The second-year observation started after the implementation of these technical changes so as to assess their effectiveness.

During the first year, 129 samples were analysed for *Legionella* and 108 for amoeba and were collected in four rounds (in May, June, September and October). After the modifications of the pipeline system and the temperature increase, 108 samples were assayed for *Legionella* over the year and 72 samples for amoeba presence and were collected in three rounds (in May, September and October).

#### Sampling

Samples from the first year were collected in four rounds, and samples from the second year were collected in three rounds after the pipeline changes.

After 10–30 s of flushing, two litres of water were collected in two sterile polystyrene bottles containing 1 ml of 3% sodium thiosulfate. Shower and tap heads were dismantled, and internal surfaces were swabbed after the water collection. The swab was then placed into the water sample collected.

The temperature of each sample was measured.

#### Legionella culture

*Legionella* detection and quantification was performed in accordance with the ISO protocol 11731:1998(E): Water quality—detection and enumeration of *Legionella*^35^.

#### Amoeba culture

A sample of 100 ml of water was filtered through a cellulose nitrate filter, 0.45 µm pore size of diameter (Millipore, Bedford, Madison). The filters were inverted on non-nutritive agar (NNA) plates seeded with inactivated *Escherichia coli* and incubated at 25 °C. The plates were microscopically monitored for outgrowth of amoeba every 3–4 days during 10 days.

#### Clinical surveillance

Active surveillance of hospital-acquired pneumonia was performed during the study period as described previously^36^. The diagnosis of *L. pneumophila* pneumonia was based on its isolation from respiratory samples and/or positive results of urinary antigen test. Cases were considered to be hospital-acquired when the patient stayed in the hospital in the 10 days preceding symptom onset.

### Data analysis

Descriptive statistics were presented as frequency counts with percentages for categorical variables. Median and 95% confidence intervals (95%CI) were calculated for quantitative variables. The comparisons between groups were performed by chi-square test in qualitative/categorical variables and the Mann–Whitney test in quantitative variables. The differences between groups were defined by p-value < 0.05.

The effect of the temperature was evaluated by establishing a cut-off of 60 °C in the water sample.
